# Characteristics of patients lost to follow-up after intravitreal anti-vascular endothelial growth factor therapy for exudative age-related macular degeneration

**DOI:** 10.1186/s12886-025-04426-9

**Published:** 2025-10-24

**Authors:** Yongwun Cho, Woong-Sun Yoo, Seong-Jae Kim, Inyoung Chung

**Affiliations:** 1 Department of Ophthalmology, Gyeongsang National University Hospital, College of Medicine, Gyeongsang National University, 79 Gangnam-ro, Jinju, 52727 Republic of Korea; 2 Health Science Institute, Gyeongsang National University, Jinju, Korea

**Keywords:** Aflibercept, Anti-VEGF, Intravitreal injections, Macular degeneration, Ranibizumab

## Abstract

**Background:**

This study aimed to analyze and compare the characteristics of patients with exudative age-related macular degeneration who were lost to follow-up after receiving intravitreal anti-vascular endothelial growth factor(anti-VEGF) injections versus those who maintained regular follow-up.

**Methods:**

This retrospective study included patients who were lost to follow-up for more than 1 year (*n* = 79) or maintained follow-up (*n* = 186) after treatment with intravitreal injections of ranibizumab or aflibercept for exudative age-related macular degeneration. Age, sex, place of residence, type of health insurance, distance from the hospital, laterality of involvement, and follow-up duration were analyzed.

**Results:**

There were no significant differences between the two groups in terms of age, sex, or type of health insurance. However, patients lost to follow-up resided significantly further from the hospital versus those with regular follow-up (33.1 ± 26.8 vs. 21.6 ± 8.5 km; *P* = 0.001). A significantly higher proportion of patients that were lost to follow-up had unilateral involvement (*P* < 0.001) and resided in rural areas (*P* < 0.001).

**Conclusion:**

Distance from the hospital, rural residency, and unilateral involvement are significantly associated with nonadherence to follow-up among patients with exudative age-related macular degeneration receiving intravitreal anti-VEGF injections.

## Introduction

Exudative age-related macular degeneration (AMD) is one of the leading causes of vision loss among the elderly population. It is characterized by choroidal neovascularization in the macula and accompanied by exudative changes such as edema and hemorrhage, resulting in progressive visual impairment. Notably, the aging population in South Korea has seen an increased prevalence of exudative AMD [1, 2].

anti-VEGF injections have become the standard treatment for exudative AMD, with many studies demonstrating its ability to significantly improve and maintain both anatomical and functional outcomes in a substantial proportion of patients [3, 4]. Some commonly used intravitreal anti-VEGF agents include bevacizumab, ranibizumab, and aflibercept. In South Korea, ranibizumab and aflibercept have been widely and interchangeably used since 2014 due to the expansion of health insurance coverage by the Ministry of Health and Welfare [5–7]. However, despite the effectiveness of anti-VEGF therapy, an increasing number of patients are lost to follow-up in clinical practice for various reasons. Recent studies have reported that patients with exudative AMD account for 22% of all cases of retinal disease that are lost to follow-up [8, 9]. Exudative AMD can result in gradual vision loss and, if left untreated, can lead to legal blindness. Therefore, it is crucial to ensure that patients adhere to follow-up visits and receive the appropriate treatment. However, in South Korea, there is a lack of research analyzing the factors that are associated with the nonadherence to follow-up among patients with exudative AMD.

This study aimed to analyze and compare the characteristics of patients with exudative AMD who were lost to follow-up after intravitreal anti-VEGF injections versus those who maintained regular follow-up. We sought to identify factors contributing to treatment discontinuation and provide insights for improving healthcare access and patient management.

## Methods

This retrospective analysis enrolled 319 patients diagnosed with exudative AMD at Gyeongsang National University Hospital from January 2017 to December 2019. These patients were registered under the rare disease registration program and received their first intravitreal anti-VEGF injection with either ranibizumab or aflibercept using the treat-and-extend regimen. The analysis excluded patients diagnosed with other ophthalmic diseases during follow-up and those that underwent ophthalmic surgeries, specifically those with a history of pars plana vitrectomy (*n* = 4), cataract surgery (*n* = 32), and other vitreoretinal diseases that could affect vision based on fundoscopy (e.g., proliferative diabetic retinopathy and retinal vein occlusion) (*n* = 18). The final analysis included 265 patients, among which 186 patients maintained regular follow-ups as of March 2024 and 79 patients were lost to follow-up for more than one year and had not returned to the hospital at the time of the investigation. The following demographic and clinical data were retrospectively collected through electronic medical records: sex, age, place of residence, type of health insurance, bilateral involvement, and duration of follow-up before loss to follow-up. Best-corrected visual acuity (BCVA) was measured at baseline, after the third injection, and at the last visit. Decimal visual acuity values were converted to logMAR units for statistical analysis. Patients with incomplete medical records were excluded, and therefore no missing data were present in the final analysis dataset. The place of residence was categorized at the city or county level. The distance from the patient’s residence to the hospital (Gyeongsang National University Hospital), defined as the shortest driving distance measured using Naver Maps, was also recorded.

For statistical analysis, categorical variables were analyzed using the chi-square test. Continuous variables were analyzed using the independent t-test for normally distributed variables or the Mann–Whitney U test for non-normally distributed variables. Statistical analyses were conducted using R software (version 4.3.1), with *P* < 0.05 considered statistically significant. The study was approved by the Institutional Review Board of Gyeongsang National University Hospital (approval No. 2023-11-019).

## Results

Among 186 patients who maintained regular follow-up, 120 (64.5%) were male and 66 (35.5%) were female, with a mean age of 74.7 ± 8.5 years. There were 156 patients (83.9%) who resided in urban areas, whereas 30 patients (16.1%) resided in rural areas. The average distance from the patient’s residences to the hospital was 21.6 ± 8.5 km. Regarding health insurance, 176 patients (94.6%) were covered by the national health insurance, while 10 patients (5.4%) were medical aid beneficiaries. Unilateral disease involvement was observed in 131 patients (70.4%), whereas bilateral involvement was seen in 55 patients (29.6%). The mean number of injections was 19.7 ± 14.8, and the mean follow-up duration was 303.4 ± 108.4 weeks.

Among 79 patients who were lost to follow-up, there were 47 (59.5%) males and 32 (40.5%) females with a mean age of 75.4 ± 9.9 years. Notably, 50 patients (63.3%) lived in urban areas, whereas 29 patients (36.7%) resided in rural areas. The average distance from their residence to the hospital was 33.1 ± 26.8 km. Regarding health insurance, 69 patients (87.3%) were covered by the national health insurance, whereas 10 patients (12.7%) were medical aid beneficiaries. All patients in this group (100%) had unilateral involvement; none had bilateral involvement. The mean number of injections was 5.0 ± 4.5, and the mean follow-up duration was 65.0 ± 63.9 weeks.

Comparing the two groups, there were no statistically significant differences in age, sex, or type of insurance coverage. In addition, BCVA was comparable between the groups at baseline, after the third injection, and at the last visit. However, patients lost to follow-up had a significantly greater distance from their residences to the hospital versus those with regular follow-up (*P* = 0.001). Additionally, patients lost to follow-up had a significantly higher proportion of those living in rural areas (*P* < 0.001) and with unilateral disease involvement (*P* < 0.001). No patient in either group experienced injection-related complications, such as endophthalmitis or retinal pigment epithelial tears, or systemic thromboembolic events, such as stroke (Table 1).

**Table 1:** Clinical characteristics of patients with exudative age-related macular degeneration

Parameter	Patients with age-related macular degeneration				*P*-value✝
	Maintained follow-up (*n* = 186) 95% CI		Lost to follow-up (*n* = 79) 95% CI		
Sex, *n* (%)					0.525
Male	120 (64.5%)	57.6–71.4	47 (59.5%)	48.7–70.3	
Female	66 (35.5%)	28.6–42.4	32 (40.5%)	29.7–51.3	
Age, years	74.7 *± 8.5*	73.5–75.9	75.4 *± 9.9*	73.2–77.6	0.521
Residence, *n* (%)					<0.001
City (urban)	156 (83.9%)	78.6–89.2	50 (63.3%)	52.7–73.9	
Gun (rural)	30 (16.1%)	10.8–21.4	29 (36.7%)	26.1–47.3	
Insurance, *n* (%)					0.072
National Health Insurance	176 (94.6%)	91.4–97.9	69 (87.3%)	78.4–93.7	
Medicaid Beneficiary	10 (5.4%)	2.1–8.6	10 (12.7%)	6.3–21.6	
Distance from patients’ residence to the hospital (km)	21.6 *± 8.5*	20.38–22.82	33.1 *± 26.8*	27.19–39.01	0.001
Eye involvement, *n* (%)					<0.001
Unilateral	131 (70.4%)	63.9–77.0	79 (100%)	100.0–100.0	
Bilateral	55 (29.6%)	23.0–36.1	0 (0%)	0.0–0.0	
Number of injections, *n*	19.7 *± 14.8*	17.57–21.83	5.0 *± 4.5*	4.01–5.99	<0.001
Follow-up period, weeks	*303.4* * ± * *108.* *4*	287.82–318.98	*65.0* * ± * *63.* *9*	50.91–79.09	<0.001
Baseline BCVA (logMAR)	0.30 ± 0.40	0.24–0.36	0.22 *± *0.40	0.13–0.31	0.433
BCVA after 3 injections (logMAR)	0.22 ± 0.40	0.16–0.28	0.22 ± 0.40	0.13–0.31	0.572
Final BCVA (logMAR)	0.30 ± 0.40	0.24–0.36	0.30 ± 0.40	0.21–0.39	0.436

✝Statistical analysis using the chi-square test, t-test, or Mann–Whitney U test, as appropriate

## Discussion

In real-world clinical settings, many patients with exudative AMD opt to discontinue intravitreal injection therapy. In our study, 79 out of 265 patients (29.8%) who received anti-VEGF injections were lost to follow-up. Previous studies by Falk et al. [10] and Vaze et al. [11] reported follow-up loss rates of 46.7% and 42.3% over 4-year and 6-year periods, respectively. Generally, higher rates of loss to follow-up are seen with longer follow-up periods. Due to the retrospective nature of this study, it is difficult to directly compare our results with those of prospective studies. Nevertheless, those that maintained follow-up in our study were able to do so for approximately 6.3 years on average, and the rate of loss to follow-up in our study (29.8%) was relatively lower than other studies with long-term follow-up. This could be because Gyeongsang National University Hospital, where this study was conducted, is the only tertiary care hospital in the province of western Gyeongnam, and no other specialized ophthalmology clinics provide treatment for AMD within a 30 km radius. Therefore, it is possible that fewer patients sought treatment at other healthcare facilities, which may have contributed to the lower follow-up loss rate observed in our study.

We chose more than 1 year as the threshold for defining lost to follow-up in accordance with previous studies [8, 12] and local clinical practice in South Korea. In this setting, patients who do not return for ≥ 1 year are administratively categorized as new patients upon re-presentation, making the one-year cutoff both clinically and administratively meaningful.Patients that were lost to follow-up resided significantly further from the hospital versus those that maintained follow-up (33.1 ± 26.8 vs. 21.6 ± 8.5 km, *P* = 0.001). Considering the location of our institution, patients living further away from the hospital were more likely to discontinue follow-up, and this may have been further worsened by the absence of alternative specialized facilities near their residences. Consistent with our results, other studies have demonstrated that patients living more than 20 miles (32.18 km) from the nearest tertiary care hospital are more likely to be lost to follow-up, especially in the absence of alternative treatment centers [8, 12]. In addition, patients residing in rural areas were more likely to be lost to follow-up than those living in urban areas. The convenience of transportation is likely a critical factor that influences follow-up adherence among rural populations. Rural areas often lack adequate public transportation systems, which can make hospital visits more challenging. Patients could also be further discouraged by the longer travel distance, as well as the associated time and financial burdens. This issue is particularly relevant among elderly or disabled patients, among whom transportation accessibility is a crucial factor in maintaining follow-up. Further research that examines the relationship between transportation convenience and follow-up loss would provide more robust evidence to support these findings.

Regarding insurance status, a greater proportion of medical aid beneficiaries was seen among those that were lost to follow-up than in those that maintained follow-up (12.7% vs. 5.4%), but this difference was not statistically significant. In contrast, in countries wherein private health insurance is predominant, studies have reported economic factors as a significant cause of treatment discontinuation [13, 14]. In South Korea, the national health insurance system covers 90% of the treatment costs for exudative AMD, which is under the rare disease registration program. This leads to a relatively small difference in treatment costs between health insurance patients and medical aid beneficiaries, which may explain why economic factors did not play a significant role in follow-up loss in our study.

In our study, patients who were lost to follow-up received substantially fewer injections and had a much shorter follow-up period than those who maintained regular visits. However, given that BCVA was comparable between groups at baseline, after the third injection, and at the last visit, these differences are more plausibly the result of discontinuation of follow-up rather than reflecting baseline disease severity. Anatomical parameters were not consistently captured, as our cohort consisted of patients with typical neovascular AMD who exhibited relatively homogeneous anatomical features, making it difficult to establish objective stratification criteria. Therefore, we relied on visual acuity as a more consistent and objective indicator of functional severity, and the absence of group differences in BCVA supports the interpretation that baseline severity was not the primary driver of loss to follow-up.

Interestingly, none of the patients that were lost to follow-up had bilateral disease involvement, and this was a significant difference compared to those who maintained follow-up. Consistent with our findings, previous studies have suggested that patients with preserved vision in one eye may not perceive the urgency of continued treatment. This causes them to discontinue treatment, even if their affected eye does not improve [8, 12, 15]. In contrast, Ehlken et al. [16] reported that bilateral disease was associated with an increased risk of discontinuation due to greater treatment burden. While our findings are more consistent with the majority of prior reports, these discrepancies highlight the need for further investigation into the role of laterality in follow-up adherence.

This study has several limitations. First, because this was a retrospective single-center study, selection bias cannot be excluded and the generalizability of the results is limited. Second, we excluded patients who received intravitreal anti-VEGF agents other than ranibizumab or aflibercept. Although these two agents were the most widely used and reimbursed in South Korea during the study period, the exclusion of bevacizumab and other agents may limit the generalizability of our findings in settings where such agents are more common. Third, IRB restrictions prevented us from directly contacting patients to determine whether they had transferred care to another institution or whether comorbidities limited their ability to return. This represents an important limitation of our study, and future prospective research should include direct patient contact or structured surveys to clarify the reasons for loss to follow-up. Finally, we focused on predefined primary variables and did not apply formal correction for multiple comparisons; therefore, the possibility of type I error cannot be excluded.Nevertheless, the strength of this study is its use of long-term follow-up data. Our results revealed that patients living further from the hospital, those residing in rural areas, and those with unilateral disease involvement were more likely to discontinue follow-up. These results are notable especially considering the scarcity of studies in South Korea focused on follow-up loss in patients with exudative AMD. Our study provides a meaningful analysis of the factors associated with follow-up loss and takes into account regional characteristics. These findings may serve as important foundational data for improving healthcare services and informing future policy development aimed at sustaining follow-up adherence in patients with exudative AMD.

Our findings suggest the need for interventions to address barriers to follow-up. During patient care, assessing residence may help identify those living far from tertiary hospitals or in rural areas, for whom measures to improve accessibility should be considered. In addition, patients with unilateral disease may underestimate the importance of continuous treatment and monitoring, highlighting the necessity of reinforced patient education. These strategies could help reduce follow-up loss and ultimately improve long-term visual outcomes.

## Data Availability

The datasets used and/or analyzed during the current study are available from the corresponding author on reasonable request.
